# Bacteriophage-Based Therapeutics for Bacterial Sexually Transmitted Infections: From Biological Barriers to Translational Strategies

**DOI:** 10.3390/pathogens15060559

**Published:** 2026-05-22

**Authors:** Nazym Syrym, Bolat Yespembetov, Sabit Kokanov, Aziz Nakhanov, Yerbol Bulatov, Azamat Abdimukhtar, Alinur Toleukhan, Yeldos Serikbay, Aibol Terebay, Aktoty Anarbekova, Kali Tileukhanov, Sabira Alpysbayeva, Makhpal Sarmykova, Bekzat Yerzhigit, Nadezhda Zinina, Marat Suleimenov, Akbope Abdykalyk

**Affiliations:** 1 Research Institute for Biological Safety Problems, Gvardeiskiy 080409, Kazakhstan; n.syrym@biosafety.kz (N.S.); b.yespembetov@biosafety.kz (B.Y.); s.kokanov@biosafety.kz (S.K.); a.nakhanov@biosafety.kz (A.N.); ye.bulatov@biosafety.kz (Y.B.); a.abdimukhtar@biosafety.kz (A.A.); a.toleukhan@biosafety.kz (A.T.); e.serikbay@biosafety.kz (Y.S.); a.terebay@biosafety.kz (A.T.); a.anarbekova@biosafety.kz (A.A.); k.tileukhanov@biosafety.kz (K.T.); s.alpysbaeva@biosafety.kz (S.A.); m.sarmykova@biosafety.kz (M.S.); b.yerzhigit@biosafety.kz (B.Y.);; 2 Department of Biotechnology and General Chemical Technology, S.D. Asfendiyarov Kazakh National Medical University, Almaty 050000, Kazakhstan; orbita_marat@mail.ru

**Keywords:** bacteriophage therapy, sexually transmitted infections, antimicrobial resistance, *Neisseria gonorrhoeae*, *Chlamydia trachomatis*, *Mycoplasma genitalium*, *Treponema pallidum*, bacterial vaginosis, endolysins, CRISPR-Cas

## Abstract

Bacterial sexually transmitted and sexually associated infections remain a major global health concern, increasingly complicated by antimicrobial resistance and the limited effectiveness of existing therapies. In this context, bacteriophage-based and phage-derived approaches have re-emerged as potential alternative antibacterial strategies. This narrative review examines their applicability across key bacterial pathogens associated with sexually transmitted infections, including *Chlamydia trachomatis*, *Neisseria gonorrhoeae*, *Mycoplasma genitalium*, *Treponema pallidum* and biofilm-associated bacterial vaginosis, with a particular focus on pathogen-specific biological barriers. Available evidence indicates that the success of phage-based interventions is strongly dependent on factors such as intracellular localisation, structural characteristics of the bacterial envelope and the presence of polymicrobial biofilms. While phage-derived platforms, including endolysins, depolymerases and engineered phages, demonstrate antibacterial activity in experimental settings, their effectiveness is uneven across different pathogens. Biofilm-associated infections appear more accessible to these approaches, whereas intracellular and structurally atypical bacteria are currently considered more challenging targets based on available mechanistic and experimental evidence. These observations highlight the need for pathogen-specific engineering strategies and delivery systems. Overall, phage-based therapeutics in this field should be considered within a framework that integrates biological constraints with targeted antimicrobial design.

## 1. Introduction

Sexually transmitted infections (STIs) remain a major global public health concern. Despite the availability of preventive measures, screening programmes, and effective treatment, more than one million new infections are acquired worldwide each day. Approximately 374 million new cases annually are caused by four major curable STIs, namely chlamydia, gonorrhoea, syphilis and trichomoniasis, among individuals aged 15–49 years [1,2].

The control of bacterial STIs is increasingly complicated by the emergence and spread of antimicrobial resistance (AMR). Among these pathogens, *Neisseria gonorrhoeae* and *Mycoplasma genitalium* have shown a marked capacity to develop resistance to multiple antimicrobial classes, thereby narrowing available treatment options [2,3,4,5]. In contrast, *Chlamydia trachomatis* remains largely susceptible to first-line therapy, although its obligate intracellular lifecycle creates inherent challenges for both diagnosis and drug delivery [5,6]. *Treponema pallidum* also remains highly susceptible to penicillin; however, reliance on a limited number of effective agents, together with the widespread emergence of macrolide resistance, highlights the need for continued surveillance and therapeutic preparedness [2,7,8,9].

Although these pathogens differ in their resistance patterns and clinical management, they share a common limitation: a restricted number of effective antimicrobial options. This constraint supports the need to explore alternative antibacterial strategies that could address multidrug resistance and reduce reliance on conventional antibiotics [10].

Against this background, bacteriophages and phage-derived antibacterial products have attracted growing interest as potential alternatives. Bacteriophages are viruses that infect bacterial cells with high specificity and may therefore serve as targeted antibacterial agents [11,12]. This selectivity may offer an important advantage over broad-spectrum antibiotics by enabling the elimination of pathogenic bacteria while preserving commensal microbiota [13,14]. However, the feasibility of phage-based approaches in STIs is strongly pathogen-dependent, since differences in bacterial structure, physiology, and infection niche directly influence whether phage-based mechanisms can be applied effectively [10].

Previous reviews have considered bacteriophage-related strategies in sexually transmitted and sexually associated infections, but their scope has remained limited. Cater et al. examined the feasibility of phage therapy in bacterial STIs, whereas Gill et al. reviewed broader biologic drug development strategies directed against major STI pathogens [10,15]. Recent advances in bacteriophage engineering and synthetic biology have significantly expanded the range of potential antibacterial strategies, including programmable phage platforms and CRISPR-Cas-based systems with enhanced specificity and resistance-targeting capabilities [16]. However, a comprehensive pathogen-specific synthesis of phage-based and phage-derived antibacterial approaches for the principal bacterial agents associated with STIs is still lacking.

Unlike previous reviews, which have primarily focused on general feasibility or broad biologic approaches, the present study introduces a pathogen-specific analytical framework that explicitly links biological barriers to mechanism-level compatibility and translational feasibility. By integrating bacterial physiology, infection niche, and antimicrobial mechanisms, this approach enables a more structured evaluation of which phage-based strategies are likely to be applicable in specific pathogen contexts. This framework also allows differentiation between pathogens that may be accessible to extracellular enzymatic or biofilm-targeting approaches and pathogens in which intracellular localisation or structural incompatibility substantially limits the applicability of conventional phage-based strategies.

This narrative review is based on a structured analysis of peer-reviewed literature retrieved from PubMed, Scopus and Web of Science. Emphasis was placed on recent experimental and translational studies, while foundational studies were included where necessary to provide mechanistic and biological context.

## 2. Major Bacterial STI Pathogens: Epidemiology, Treatment Limitations and Antimicrobial Resistance

**Figure 1:**
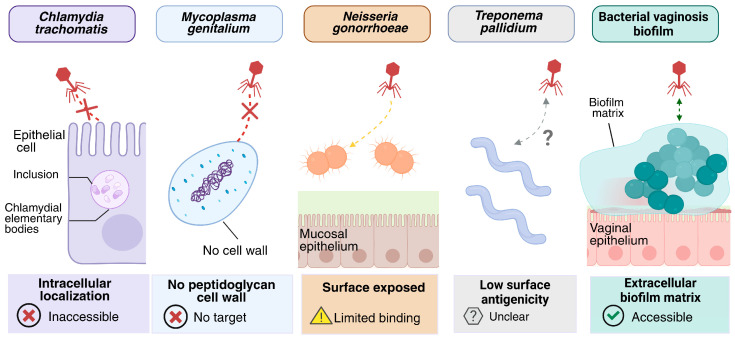
Pathogen-specific biological barriers influencing the applicability of phage-based antibacterial strategies in sexually transmitted and sexually associated infections. Barriers are categorised as biological exclusion (e.g., intracellular localisation), structural limitation (e.g., absence of peptidoglycan), and target accessibility constraints, reflecting differences in therapeutic accessibility, engineering feasibility and translational potential across pathogen groups.

Major bacterial sexually transmitted pathogens differ in prevalence, age distribution, asymptomatic carriage, clinical presentation, and long-term outcomes. These differences shape their public health impact and influence diagnosis, treatment, and control strategies. These pathogen-specific differences reflect distinct biological and structural constraints that influence therapeutic accessibility, as illustrated in Figure 1. Key resistance mechanisms and dominant biological constraints are summarised in Table 1. Dominant biological constraints do not represent mutually exclusive barriers, and several pathogens exhibit overlapping structural, ecological and translational limitations. The WHO Global Health Sector Strategies on HIV, viral hepatitis, and sexually transmitted infections 2022–2030 set ambitious targets for reducing STI incidence and emphasize expanded access to testing, partner services, and the development of affordable diagnostics, treatments, and vaccines [17].

### 2.1. Chlamydia trachomatis

*Chlamydia trachomatis* remains the most frequently reported bacterial STI worldwide and continues to disproportionately affect adolescents and young adults. An estimated 128.5 million new infections occur annually among individuals aged 15–49 years, with a prevalence of 4.0% in women and 2.5% in men [2].

A defining epidemiological feature of *C. trachomatis* infection is the high proportion of asymptomatic cases, which facilitates silent transmission and delayed diagnosis. Up to 70–80% of infections in women and approximately 50% in men are asymptomatic, with the highest burden observed in individuals aged 15–24 years [7,18].

Clinically significant antimicrobial resistance in *C. trachomatis* remains uncommon, and stable population-level resistance has not been clearly demonstrated [19,20]. This pattern is partly explained by its obligate intracellular lifecycle, which complicates antimicrobial susceptibility testing and limits routine resistance surveillance [6,20,21].

Sporadic isolates with reduced susceptibility to macrolides and tetracyclines have been reported; however, these findings remain rare and inconsistent and do not indicate widespread clinical resistance [22,23,24]. Proposed resistance-associated mechanisms include mutations in the 23S rRNA gene and alterations in *gyrA* and *parC*, although these observations are based on limited data and their clinical relevance remains uncertain [20,25].

In contrast to *Chlamydia suis*, where stable tetracycline resistance is well established, resistance in *C. trachomatis* appears sporadic and not widely disseminated [6,20]. In addition, the absence of standardized susceptibility testing methods and clinical interpretive criteria complicates the assessment of treatment failure and limits surveillance efforts [20,26]. The main challenge in managing *C. trachomatis* infection is therefore not classical antimicrobial resistance, but asymptomatic carriage, persistence, reinfection and underdiagnosis [7].

### 2.2. Neisseria gonorrhoeae

*Neisseria gonorrhoeae* remains one of the most clinically significant bacterial STIs due to its high global burden and its exceptional capacity to develop antimicrobial resistance. Approximately 87 million new infections occur annually worldwide [2]. A substantial proportion of infections, particularly in women, are asymptomatic, contributing to ongoing transmission.

Recent surveillance data from Europe indicate a continued increase in incidence. In 2023, 96,969 confirmed cases were reported in the EU/EEA, corresponding to a notification rate of 25.0 per 100,000 population. This represents a 31% increase compared with 2022 and the highest level recorded since surveillance began in 2009 [27].

*N. gonorrhoeae* represents a clear example of rapid antimicrobial adaptation. Over time, it has developed resistance to sulfonamides, penicillins, tetracyclines, macrolides, fluoroquinolones and earlier-generation cephalosporins, progressively reducing available treatment options. Ceftriaxone remains the cornerstone of current therapy; however, decreasing susceptibility and the emergence of ceftriaxone-resistant and extensively drug-resistant strains are of increasing concern [3,4,28,29,30].

Global surveillance data from 2019 to 2022 confirm widespread resistance to azithromycin and persistently high resistance to ciprofloxacin. Reduced susceptibility to ceftriaxone and cefixime has also been reported in multiple regions, particularly in the WHO Western Pacific Region [28].

Resistance evolution in *N. gonorrhoeae* is driven by high genetic plasticity, including horizontal gene transfer and recombination affecting key determinants such as mosaic *penA* alleles and efflux pump regulation [3].

In addition to urogenital infection, *N. gonorrhoeae* frequently colonises extragenital anatomical sites, particularly the pharynx and rectum, which may serve as persistent reservoirs contributing to asymptomatic transmission and treatment failure [7,27,29]. These niches differ substantially in mucosal composition, microbial ecology, immune exposure, and biofilm formation, potentially influencing the accessibility and stability of phage-based interventions [31,32]. Pharyngeal colonisation is of particular concern because it may facilitate prolonged persistence and horizontal genetic exchange with commensal *Neisseria* species, thereby contributing to antimicrobial resistance evolution [3,29]. These site-specific biological differences further complicate the translational feasibility of phage-based therapeutic strategies against gonococcal infection.

### 2.3. Mycoplasma genitalium

*Mycoplasma genitalium* has emerged as an important sexually transmitted pathogen over the past two decades. Although less prevalent than chlamydia or gonorrhoea, it is clinically significant due to its association with non-gonococcal urethritis in men and cervicitis and pelvic inflammatory disease in women [33,34,35].

The organism is detected in approximately 1–3% of the general population, with prevalence increasing to 10–20% in high-risk groups and among individuals with non-gonococcal urethritis [34,35,36,37]. It is responsible for approximately 15–20% of non-gonococcal urethritis, 20–25% of non-chlamydial urethritis, and up to 40% of persistent or recurrent urethritis [7,35].

From a therapeutic perspective, *M. genitalium* presents a major challenge because it lacks a peptidoglycan cell wall, rendering β-lactam antibiotics ineffective. Treatment therefore relies primarily on macrolides and fluoroquinolones. However, resistance to both classes has increased markedly in recent years. Macrolide resistance frequently exceeds 50–60% and may reach over 80% in certain populations, while fluoroquinolone resistance and dual resistance are also increasing [37,38,39,40].

Macrolide resistance is mainly associated with mutations in the 23S rRNA gene at positions 2058 and 2059, whereas fluoroquinolone resistance is linked to mutations in the quinolone resistance-determining region of *parC*, particularly at residues S83 and D87 [41,42]. The widespread use of azithromycin, especially single-dose regimens in syndromic management, has been identified as a key driver of resistance selection [35,38].

The accumulation of resistance-associated mutations has led to the emergence of multidrug-resistant strains associated with treatment failure and persistent infection [37,39,41]. Current management increasingly relies on resistance-guided and sequential therapy, combined with test-of-cure strategies [7,35,38].

### 2.4. Treponema pallidum

*Treponema pallidum*, the causative agent of syphilis, remains a major global public health concern, with approximately 6–7 million new infections annually among adults aged 15–49 years [2]. The disease progresses through distinct clinical stages and may lead to severe systemic complications if untreated [43,44].

Recent data indicate increasing incidence in multiple regions. In the EU/EEA, 41,051 cases were reported in 2023, representing a 13% increase compared with 2022 and approximately a twofold increase since 2014 [45]. Congenital syphilis remains a major concern, with adverse outcomes occurring in 50–80% of untreated maternal infections [44,46].

Unlike other major bacterial STIs, *T. pallidum* remains highly susceptible to penicillin, which continues to be the first-line treatment. No confirmed clinical resistance to penicillin has been demonstrated [7,43]. This sustained susceptibility is likely related to its reduced genome and limited capacity for horizontal gene transfer [47].

In contrast, macrolide resistance is well established and is primarily associated with point mutations in the 23S rRNA gene, particularly A2058G and A2059G, which are strongly linked to treatment failure [7,8,48]. The prevalence of macrolide-resistant strains exceeds 50% in some regions, limiting their clinical utility [8,49,50].

### 2.5. Bacterial Vaginosis and Gardnerella-Associated Biofilms

Bacterial vaginosis (BV) is not traditionally classified as an STI but is strongly associated with sexual activity and is relevant within the broader context of sexually associated infections. It is the most common vaginal disorder among women of reproductive age, with global prevalence ranging from approximately 23% to 29% [51,52,53].

BV is characterized by structured polymicrobial biofilms adherent to the vaginal epithelium. These biofilms are typically dominated by *Gardnerella* spp. and include other anaerobic organisms such as *Atopobium vaginae*, *Sneathia* spp., *Mobiluncus* spp., and *Prevotella* spp. [54,55,56,57,58,59,60].

Biofilm formation involves adhesion, colonization, extracellular matrix production, and dispersal, supporting long-term persistence and resistance to treatment. Reduced antimicrobial efficacy is primarily associated with biofilm-related tolerance, limited drug penetration, and metabolic heterogeneity rather than classical resistance mechanisms [61,62,63].

Recurrence rates remain high, with more than 50% of cases recurring within 6–12 months and up to 70–80% within one year [62,64,65]. This is linked to incomplete biofilm eradication and failure to restore a stable Lactobacillus-dominated microbiota [62,66].

Recent studies indicate that phage-derived enzymes, including endolysins, can disrupt *Gardnerella*-dominated biofilms and restore antimicrobial susceptibility [67]. The extracellular nature of these biofilms may make them more accessible to phage-derived approaches, highlighting their potential as therapeutic targets. However, targeting *Gardnerella* alone may not be sufficient for durable clinical resolution, as recurrence is strongly associated with failure to restore a stable Lactobacillus-dominated microbiota. This highlights the need for combination approaches integrating biofilm disruption with microbiome restoration. These features are consistent with the broader patterns observed across STI-associated pathogens, as summarized in Table 1.

**Table 1:** Major bacterial sexually transmitted pathogens: key resistance mechanisms and treatment challenges.

Pathogen	Burden	Key Clinical Features	AMR Status	Main Resistance Mechanisms	Dominant Biological Constraint	Major Treatment Limitations
*Chlamydia trachomatis*	127–128 M/year [2]	Often asymptomatic; may lead to PID and infertility [7,18,25]	Low (rare documented resistance) [19,20,23]	Putative mutations in 23S rRNA, *rplD*, *gyrA/parC* [6,20,23]	Obligate intracellular [6]	Limited resistance surveillance; persistence and reinfection [7,19,20]
*Neisseria gonorrhoeae*	~87 M/year [2]	Urethritis, cervicitis, PID; often asymptomatic in women [7,29]	High (increasing) [3,4,28,29]	Mosaic *penA* alleles; *mtrR*-mediated efflux [3,29]	High genetic plasticity and antigenic variation [28]	MDR/XDR strains; narrowing treatment options [27,28]
*Mycoplasma genitalium*	~1–3% in general population; higher in high-risk groups [34,36]	Persistent urethritis and cervicitis [33,34]	Very high [35,37,38,39]	23S rRNA mutations (A2058G/A2059G); *parC* mutations [37,38,39,42]	Absence of cell wall [33]	Limited therapeutic options; high resistance burden [35,37,38,39]
*Treponema pallidum*	6–7 M/year [2]	Multistage systemic infection; congenital infection [44,46]	Low overall; macrolide resistance present [8,48]	23S rRNA mutations (A2058G, A2059G) [8,48]	Non-cultivable/experimentally intractable [47]	Reliance on penicillin; limited alternatives [7,43]
Bacterial vaginosis (*Gardnerella*-associated)	23–29% [51,53]	Vaginal discharge; high recurrence [62,65]	Functional tolerance (biofilm-associated) [61,62,63]	Polymicrobial biofilm formation; sialidase activity [55,58,60]	Structured polymicrobial biofilm adherent to vaginal epithelium [54,55,56]	High recurrence despite therapy; frequently associated with biofilm persistence and microbiome instability [62,64,65,66,67,68,69]

## 3. Phage-Based and Phage-Derived Antimicrobial Strategies

The renewed interest in bacteriophage-based antimicrobials is driven not only by the global expansion of antimicrobial resistance but also by the recognition that conventional antibiotics are often poorly adapted to the biological complexity of infection niches, particularly in mucosal and polymicrobial environments [12,17,70]. Rather than representing a single therapeutic modality, phage-based approaches comprise a heterogeneous group of platforms that differ fundamentally in their mechanisms of action, interaction with bacterial targets, and dependence on host and environmental factors [12,71,72].

**Figure 2:**
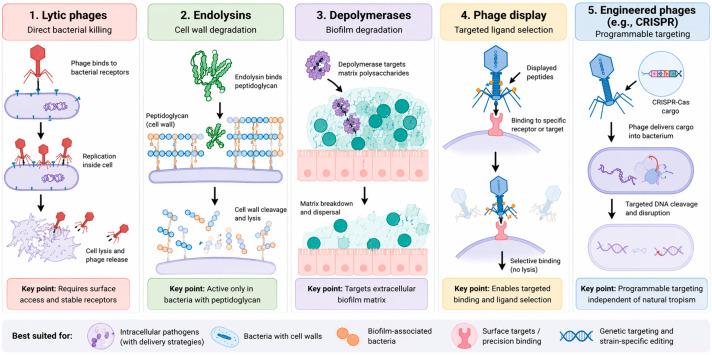
Overview of major bacteriophage-based and phage-derived antimicrobial strategies and their mechanisms of action. The figure includes replicating (lytic phages), enzymatic (endolysins, depolymerases), targeting (phage display-derived ligands), and programmable (engineered phages and CRISPR-Cas systems) platforms. Evidence maturity varies substantially between platforms and pathogens, ranging from conceptual rationale to experimental validation.

From a functional perspective, these approaches can be broadly classified into four categories: replicating systems, represented by lytic bacteriophages; enzymatic systems, including endolysins and depolymerases; targeting platforms, such as phage display-derived ligands; and programmable systems, including engineered phages and CRISPR-Cas-based antimicrobials [71,73,74,75,76]. This classification reflects not only mechanistic differences but also distinct constraints related to delivery, target accessibility and compatibility with bacterial physiology. In bacterial sexually transmitted and sexually associated infections, the relevance of these platforms is shaped by shared ecological and biological features, including colonization of mucosal surfaces, interaction with complex microbial communities, and, in some cases, intracellular localization of pathogens [10,12]. The major phage-based and phage-derived antibacterial platforms and their mechanisms of action are summarized in Figure 2.

### 3.1. Lytic Bacteriophages

Lytic bacteriophages represent the classical form of phage-based antibacterial therapy. Their defining feature is the ability to infect susceptible bacteria, replicate intracellularly, and release progeny virions through host cell lysis, thereby amplifying antibacterial activity at the site of infection [12,77,78]. This self-propagating behavior distinguishes phages from conventional antibiotics, whose effective concentration typically declines over time. Temperate phages are generally considered unsuitable for therapeutic applications because of the risk of horizontal gene transfer and lysogenic conversion [79].

A key advantage of lytic phages is their high specificity, often at the strain level, which enables targeted elimination of pathogenic bacteria while minimizing disruption of commensal microbiota [80]. However, this specificity also limits therapeutic coverage and frequently necessitates the use of phage cocktails or individualized phage selection strategies [13,81].

Phages can also interact with biofilms, where they diffuse through water channels and replicate within bacterial microcolonies [82,83]. In some cases, phage-associated enzymes contribute to degradation of extracellular matrix components, facilitating deeper penetration into structured communities [70]. Experimental studies have consistently demonstrated reductions in bacterial load and biofilm biomass following phage treatment under in vitro and preclinical conditions, although complete eradication is rarely achieved without combination approaches [78,84].

Despite these advantages, the applicability of lytic phages to sexually transmitted infections is constrained by pathogen-specific biological factors. Intracellular localization, as observed in *Chlamydia trachomatis*, severely limits access of phage particles to bacterial cells [6]. In addition, adsorption requires stable surface receptors, which may be absent or highly variable in certain pathogens [80,85]. Gram-negative bacteria introduce an additional barrier through the outer membrane, which can restrict access to receptor sites and reduce infection efficiency [85].

Resistance to phages can arise through multiple mechanisms, including receptor modification, restriction-modification systems, and CRISPR-mediated immunity [85,86]. Host immune responses, including the development of neutralizing antibodies, may further reduce phage persistence in vivo, particularly in mucosal environments characterized by active immune surveillance and rapid clearance [87,88,89]. Together with challenges related to delivery, stability and regulatory standardization, these factors limit the clinical translation of lytic phage therapy in this context [12,90,91]. Overall, lytic phages are most plausibly applicable in extracellular infections with accessible and sufficiently stable bacterial targets, whereas their applicability appears limited in intracellular pathogens and organisms with unstable or poorly accessible surface receptors.

### 3.2. Endolysins and Lysin-Based Systems

Endolysins are bacteriophage-encoded peptidoglycan hydrolases responsible for host cell lysis during the final stage of the phage replication cycle [71,73,92]. When applied externally as purified proteins, they act as direct antibacterial agents independent of phage replication, allowing their use in a manner analogous to conventional antimicrobials [93].

Most endolysins exhibit a modular structure consisting of catalytic domains and, in many cases, cell wall-binding domains that contribute to substrate specificity [94]. This structural organization enables selective antibacterial activity and may reduce off-target effects on commensal microbiota [95]. Resistance appears to arise less readily than with many conventional antibiotics, likely because lysins target conserved structural components of the bacterial cell wall [73,96,97].

The antibacterial activity of endolysins is generally strongest against Gram-positive bacteria, where peptidoglycan is directly accessible. In Gram-negative organisms, the outer membrane acts as a major permeability barrier, substantially limiting enzyme access to the cell wall [73,98,99]. To overcome this limitation, engineered lysins such as artilysins have been developed by combining lytic domains with membrane-disrupting peptides, thereby extending activity toward Gram-negative targets [98,100]. Importantly, however, lysin-based activity still depends on the presence of accessible peptidoglycan. Consequently, these systems are largely incompatible with wall-less organisms such as *Mycoplasma genitalium* and are unlikely to be effective in obligate intracellular settings where enzyme access is severely restricted.

Within sexually associated infections, lysin-based approaches are most relevant in extracellular and biofilm-associated conditions. A notable example is bacterial vaginosis, where polymicrobial biofilms dominated by *Gardnerella* spp. contribute to persistence and recurrence [54,62,64,66]. In this setting, the engineered endolysin PM-477 has demonstrated selective activity against *Gardnerella*, effective biofilm disruption, and minimal impact on beneficial lactobacilli [101].

However, lysin-based systems face several practical limitations, including susceptibility to proteolytic degradation, limited persistence in vivo, short local half-life, and challenges in achieving effective delivery and retention at mucosal surfaces [12]. Their translational potential in genital tract infections will therefore depend not only on antibacterial potency, but also on formulation strategy and local pharmacological stability.

### 3.3. Depolymerases and Antibiofilm Enzymes

Phage-encoded depolymerases are enzymes that degrade extracellular polysaccharide structures such as bacterial capsules and biofilm matrix components, thereby facilitating access to the bacterial cell surface [74,102,103]. Because many clinically important bacteria rely on these extracellular structures for persistence and immune evasion, depolymerases have also attracted attention as stand-alone antivirulence and antibiofilm agents [72,74]. Their relevance in sexually associated infections is therefore primarily linked to conditions in which extracellular matrix architecture plays a central role in pathogenesis.

These enzymes are structurally and functionally diverse. Depending on substrate specificity, they may target capsular polysaccharides, lipopolysaccharide-associated glycans, or extracellular polymeric substances within biofilms. Functionally, they include both hydrolases and polysaccharide lyases, reflecting the heterogeneity of carbohydrate-rich bacterial surface structures [74,102].

Unlike endolysins, which directly hydrolyse peptidoglycan and rapidly lyse bacterial cells, depolymerases act primarily by dismantling extracellular protective barriers. Their antibacterial effect is therefore largely indirect and is mediated through improved access of host immune factors [103], enhanced serum-mediated killing, and increased penetration or efficacy of co-administered antimicrobial agents [74]. This distinction is important, as depolymerases should not be regarded as broadly bactericidal equivalents of lysins but rather as matrix-disrupting adjuncts.

This mechanism is particularly relevant in biofilm-associated infections. Experimental studies have shown that depolymerase treatment can reduce biofilm biomass and enhance antimicrobial efficacy, although complete eradication is uncommon without combination approaches [13,103]. Accordingly, the strongest current rationale for depolymerase-based strategies lies in their use in combination with antibiotics, lysins, phages or host immune mechanisms, rather than as stand-alone curative interventions.

Among sexually associated conditions, bacterial vaginosis represents the most plausible application of this strategy, as its extracellular biofilm matrix provides an accessible and structurally defined target [54,62,64,66]. In this context, matrix-disrupting approaches are mechanistically attractive, as disruption of the extracellular scaffold may enhance the penetration and efficacy of companion therapeutics. However, direct STI-specific evidence remains limited, and most available data are derived from non-genital model systems.

Overall, depolymerase-based strategies appear better suited to extracellular, biofilm-driven conditions than to intracellular sexually transmitted pathogens. Their future clinical value in the genital tract will likely depend on combination use with antibiotics, lysins, phages, or host immune mechanisms, together with adequate mucosal stability and retention.

### 3.4. Phage Display and Targeting Technologies

Phage display is a ligand selection technology that enables the identification of peptides or proteins with high affinity and specificity toward defined targets [75,104,105]. In antimicrobial research, its primary value lies in the generation of targeting molecules rather than direct bacterial killing [106].

A key advantage of phage display is the ability to perform whole-cell biopanning, preserving the native structure of bacterial surface targets and enabling selection of ligands against physiologically relevant epitopes [107,108,109]. These ligands can be incorporated into a range of therapeutic platforms, including peptide–drug conjugates, antimicrobial peptides, antibody fragments, and nanoparticle-based delivery systems [106].

Such approaches enable targeted delivery of antimicrobial agents and may improve efficacy while minimizing disruption of commensal microbiota. In addition, targeting ligands can function as anti-virulence agents by interfering with bacterial adhesion, colonization, metabolism, or biofilm formation.

In sexually transmitted infections, phage display-based systems are therefore particularly relevant for pathogens in which classical phage therapy is constrained, including intracellular organisms such as *Chlamydia trachomatis* and antigenically variable species such as *Neisseria gonorrhoeae* [6,31,110]. However, most applications remain at an early translational stage and are primarily supported by in vitro or proof-of-concept studies rather than clinically validated infection models [12,111,112].

### 3.5. Engineered Phage Systems and CRISPR-Cas Platforms

Advances in synthetic biology have enabled the development of engineered phage systems that extend beyond natural lytic activity toward programmable antimicrobial platforms [72,76,113,114]. These approaches use bacteriophages or phage-derived vectors to deliver functional payloads, most notably CRISPR-Cas systems, into target bacteria.

CRISPR-based antimicrobials function through sequence-specific recognition of bacterial DNA, resulting in targeted genome cleavage or selective elimination of resistance and virulence determinants [115]. Experimental studies have demonstrated that phage-delivered CRISPR systems can reduce bacterial viability and restore antibiotic susceptibility under controlled conditions [76,113,114,115,116,117].

A major strength of CRISPR-based antimicrobials is their precision. However, their efficacy depends critically on efficient delivery, including adsorption, entry of the genetic payload, and sufficient intracellular expression of the effector system [12,118,119]. These constraints are particularly relevant in sexually transmitted infections, where intracellular pathogens and structurally atypical bacteria may limit accessibility.

At present, no clinically validated CRISPR-based antimicrobial strategy has been established for major bacterial STI pathogens, and most available data remain at the proof-of-concept stage [120]. Their future clinical relevance will depend on overcoming challenges related to delivery, stability, host range, and regulatory acceptance.

Although these platforms offer diverse antibacterial mechanisms, their practical applicability in sexually transmitted and sexually associated infections varies substantially depending on pathogen-specific biological and structural factors, which are examined in the following section.

Across these platforms, the strength of available evidence varies considerably. In general, the data range from direct pathogen-specific experimental studies to proof-of-concept findings in model systems, and in some cases only to mechanistic or theoretical rationale without direct validation. Recognizing these differences is important when interpreting translational potential, as several approaches appear biologically plausible but have not yet been confirmed in pathogen-specific experimental settings.

## 4. Biological Constraints That Determine the Feasibility of Phage-Based Therapeutics

The following section examines how pathogen-specific biological constraints shape the feasibility of the phage-based strategies outlined above. The limited progress of phage-based therapeutics in STI-associated pathogens reflects multiple biological constraints related to pathogen accessibility, structural compatibility, and infection niche [10,12].

Importantly, these constraints often coexist and interact rather than acting as isolated limitations. Their relative impact also differs in the level of supporting evidence, ranging from experimentally validated mechanisms to indirect or conceptual inference, as summarised in Table 2. The central question is therefore not simply whether a phage-based approach exists, but whether it can access the target organism, engage a sufficiently stable molecular interface, and retain activity under clinically relevant conditions.

### 4.1. Intracellular Sequestration in Chlamydia trachomatis: When Access Becomes the Primary Barrier

Among major bacterial sexually transmitted pathogens, *Chlamydia trachomatis* presents one of the most fundamental barriers to phage-based intervention due to its obligate intracellular lifecycle [6]. The organism replicates within a host-derived inclusion and alternates between extracellular elementary bodies and intracellular reticulate bodies, a developmental cycle that severely restricts access of extracellular antibacterial agents [6,18,25].

This intracellular localisation creates a direct mismatch with classical lytic phage therapy, which depends on adsorption to accessible bacterial surfaces and productive infection of target cells [12,85]. The same constraint also limits lysin-based approaches. Although endolysins do not require phage replication, they still depend on physical access to bacterial peptidoglycan [71,73]. In *C. trachomatis*, potential structural targets are effectively shielded by the intracellular niche, making delivery, rather than catalytic activity, the primary limitation.

This barrier does not fully exclude phage-enabled strategies but shifts attention toward non-lytic approaches that decouple targeting from direct bacteriolysis. Phage display-derived ligands and engineered delivery systems may be conceptually compatible with chlamydial biology, as they could be adapted for host-cell targeting, receptor-mediated uptake, or interference with host–pathogen interactions [75,76,104,105,106,113,114,115]. However, such applications remain largely theoretical, and no clinically relevant phage-based platform has yet been established for intracellular chlamydial infection.

Thus, *C. trachomatis* represents a pathogen in which intracellular localisation is likely to constitute a major constraint for classical phage therapy. The principal limitation is not the absence of antibacterial strategies, but the limited compatibility between these strategies and the intracellular infection niche.

### 4.2. Structural Incompatibility in Mycoplasma genitalium: When the Canonical Enzymatic Target Is Absent

A distinct and more intrinsic constraint is observed in *Mycoplasma genitalium*. Unlike *C. trachomatis*, where intracellular localisation primarily limits accessibility, the major barrier in *M. genitalium* is structural: the absence of peptidoglycan eliminates the essential substrate required for endolysins and related cell wall-degrading enzymes [33,34]. This renders lysin-based approaches largely incompatible with the organism based on current mechanistic understanding.

This limitation parallels the well-established intrinsic resistance of mycoplasmas to beta-lactam antibiotics, which also depend on peptidoglycan synthesis and cell wall integrity [33,38]. Even if delivery could be achieved, enzymatic activity would remain ineffective in the absence of a suitable substrate. The constraint is therefore mechanistic rather than pharmacological.

The clinical relevance of this incompatibility is amplified by the increasing prevalence of antimicrobial resistance in *M. genitalium*. Macrolide resistance is widespread, and fluoroquinolone resistance is rising globally [37,38,39,41,42,121]. The emergence of multidrug-resistant lineages further emphasises the need for alternative therapeutic strategies, while also illustrating that not all phage-derived systems are biologically feasible.

In this context, non-lytic approaches may offer greater compatibility. Phage display technologies can identify ligands targeting membrane-associated structures, and engineered delivery systems may be adapted to interfere with adhesins or other virulence-associated factors [75,104,105,106]. Although such strategies remain experimental, they are more consistent with *M. genitalium* biology than approaches dependent on peptidoglycan degradation.

### 4.3. Outer Membrane Exclusion and Receptor Instability in Neisseria gonorrhoeae: A Dual-Interface Barrier

The therapeutic challenge in *Neisseria gonorrhoeae* reflects a combination of structural and biological constraints. As a Gram-negative organism, it possesses an outer membrane that restricts access of externally applied lytic enzymes to the underlying peptidoglycan layer, thereby limiting the activity of native endolysins and related hydrolases [29,73,98]. This restricts lysin-based strategies unless membrane-penetrating constructs, such as engineered artilysins, can be effectively adapted [98,99,100].

A second and equally important barrier is receptor instability. *N. gonorrhoeae* exhibits extensive variation in surface structures involved in colonisation and immune evasion, including pili, opacity-associated proteins, and lipooligosaccharides [3,29,31]. Because many phage-based strategies rely on stable surface receptors for binding, this variability reduces targeting reliability and increases the risk of therapeutic escape.

Although phage–host interactions have been described in the gonococcal lineage, including filamentous systems such as NgoΦ6, this does not directly translate into therapeutic feasibility [32]. The same surface plasticity that supports immune evasion also complicates receptor-dependent targeting.

This issue is particularly relevant in the context of antimicrobial resistance, as *N. gonorrhoeae* has acquired resistance to nearly all major antibiotic classes [4,27,28,29,30]. However, the biological features that enable adaptation and persistence simultaneously limit the effectiveness of phage-based approaches. Overall, current evidence remains limited and largely indirect, and further pathogen-specific validation is required.

### 4.4. Treponema pallidum: Biological Plausibility Under Conditions of Experimental Inaccessibility

Evaluation of phage compatibility in *Treponema pallidum* is complicated by fundamental limitations in experimental accessibility. Unlike other bacterial pathogens, *T. pallidum* has historically been difficult to cultivate and manipulate in vitro, restricting direct investigation of phage interactions [44,47,48,49,50,122].

Its outer membrane contains a low density of surface-exposed proteins, a feature associated with immune evasion and persistent infection. From a phage perspective, this may limit the availability of accessible receptors required for adsorption. In addition, the organism grows slowly and depends strongly on host-associated conditions, further complicating experimental analysis.

Indirect support for phage–spirochete interactions remains limited and largely theoretical. Although bacteriophage-based strategies cannot be excluded in principle, there is currently no direct evidence supporting their applicability within the genus Treponema. However, these observations cannot be directly extrapolated to *T. pallidum* due to substantial differences in genome structure, ecological niche and host dependence. Accordingly, while such considerations support general biological plausibility, they do not provide direct evidence of phage susceptibility in *T. pallidum*.

Recent advances, including the development of long-term culture systems and initial genetic manipulation approaches, have begun to improve experimental tractability [123,124]. However, these developments do not yet establish the feasibility of phage-based therapy in this organism.

From a translational perspective, evaluation of phage compatibility in *T. pallidum* will require several key experimental milestones, including identification of potential phage–host interactions, development of stable in vitro cultivation systems, and establishment of genetic tools enabling receptor characterisation and susceptibility testing. Until such advances are achieved, conclusions regarding phage applicability should be considered preliminary.

### 4.5. Biofilm-Associated Bacterial Vaginosis: When the Barrier Becomes the Therapeutic Target

Bacterial vaginosis represents a distinct therapeutic context. Unlike intracellular pathogens or structurally atypical bacteria, the primary barrier is the presence of a structured polymicrobial biofilm adherent to the vaginal epithelium [51,52,53,54,55,56,57,58,59,60,61,62,63,64,65,66,67,69,101,125,126,127]. These biofilms, typically dominated by *Gardnerella* spp., are closely associated with persistence, reduced antimicrobial susceptibility, and high recurrence rates [54,62,64,66].

This feature fundamentally alters the therapeutic landscape. In contrast to other STI pathogens, the barrier in BV is extracellular and physically accessible. The biofilm matrix itself becomes a viable therapeutic target, particularly for phage-derived enzymatic approaches.

Depolymerases can degrade matrix-associated polysaccharides, while endolysins may act on exposed bacterial cells following partial disruption of biofilm architecture [101,102,103]. This creates a scenario in which the structural barrier to treatment also defines the point of therapeutic intervention.

The engineered endolysin PM-477 provides a key example, demonstrating selective activity against *Gardnerella* spp. and effective disruption of BV-associated biofilms while sparing beneficial lactobacilli [101].

However, targeting *Gardnerella* alone may not be sufficient for durable clinical resolution, as recurrence is strongly associated with failure to restore a stable Lactobacillus-dominated microbiota. This highlights the importance of combination strategies integrating biofilm disruption with microbiome restoration.

Despite these promising findings, several challenges remain, including formulation stability, mucosal retention, and the predominance of preclinical evidence.

Nevertheless, compared with intracellular or structurally constrained pathogens, BV represents one of the most promising targets for phage-derived therapeutic strategies. A comparative overview of phage-based and phage-derived strategies across major bacterial sexually transmitted and sexually associated infections is provided in Table 2.

**Table 2:** Phage-based and phage-derived antimicrobial strategies in STI-associated pathogens: mechanisms, representative studies, and evidence level.

Target Organism	Phage-Related Strategy	Experimental System	Key Findings	Dominant Constraint	Evidence Level	Reference
*Gardnerella* spp.	Engineered endolysin PM-477	Ex vivo vaginal biofilms; in vitro single- and dual-species biofilms	Selective elimination of *Gardnerella* spp. with preservation of lactobacilli; disruption of BV-associated biofilms	Biofilm (accessible extracellular target)	Experimental (preclinical)	[101,125]
BV-associated polymicrobial biofilms	Phage-derived antibiofilm enzymes (endolysins, depolymerases)	In vitro and ex vivo biofilm models	Enzymatic disruption of extracellular matrix and enhanced biofilm clearance; however, polymicrobial structure and recurrence remain key challenges	Biofilm matrix (extracellular accessibility)	Experimental (preclinical)	[72,73,74,102,103,128,129]
*Chlamydia trachomatis*	Phage display-derived targeting ligands; engineered delivery systems	Conceptual/platform-level rationale based on intracellular infection biology and mucosal delivery constraints	Intracellular localisation likely limits classical phage therapy; delivery-oriented strategies may be more compatible, but no pathogen-specific therapeutic platform has been experimentally validated	Intracellular localisation	Conceptual	[6,75,104,105,106,111,112]
*Neisseria gonorrhoeae*	Filamentous bacteriophage NgoΦ6; gonococcal phage/prophage-based systems	Genomic and experimental studies	Evidence of phage–host interaction supports biological plausibility; however, no validated therapeutic application has been demonstrated, and receptor variability may limit targeting stability	Outer membrane barrier and receptor variability	Indirect (preclinical)	[32,130]
*Mycoplasma genitalium*	Phage display-derived targeting ligands; non-lytic delivery-oriented systems	Conceptual/platform-level rationale based on wall-less cell biology and surface-targeting strategies	Absence of peptidoglycan renders lysin-based approaches mechanistically incompatible; alternative targeting strategies are conceptually plausible but remain unvalidated	Lack of peptidoglycan	Conceptual	[33,34,75,104,105,106]
*Treponema pallidum*	Not established; inferred from recent cultivation and genetic tools	In vitro cultivation and early genetic engineering studies	No bacteriophage has been identified and therapeutic feasibility remains untested despite recent advances in cultivation and genetic manipulation	Experimental inaccessibility	Unknown/low evidence	[123,124]

## 5. Discussion: Translational Outlook and Clinical Challenges

The increasing prevalence of antimicrobial resistance in bacterial sexually transmitted infections has intensified interest in alternative therapeutic strategies. However, clinical translation remains limited by multiple biological and delivery-related constraints across STI-associated pathogens. These differences in feasibility reflect varying levels of supporting evidence across strategies.

Clinical translation therefore depends not only on identifying effective phage-derived agents, but also on developing delivery systems tailored to the anatomical and physicochemical conditions of the genital tract [12,111,112]. In this context, delivery rather than intrinsic antibacterial activity likely represents the primary bottleneck for clinical implementation.

Effective delivery to mucosal surfaces remains a central challenge. Sexually transmitted pathogens inhabit dynamic environments characterised by continuous fluid turnover, mucus barriers, and heterogeneous epithelial architecture [111,131], all of which limit retention and local bioavailability of therapeutic agents. Conventional topical formulations, such as vaginal gels, typically provide only transient exposure [132], whereas intravaginal rings, mucoadhesive formulations and nanoparticle-based systems may enable more sustained release and improved mucosal coverage [112,131,133,134].

Clinical implementation is also highly dependent on anatomical site. The vaginal, cervical, urethral, rectal, and pharyngeal environments differ substantially in epithelial structure, mucus composition, immune activity, and physicochemical conditions. The vaginal niche, for example, is characterised by acidic pH and a Lactobacillus-dominated microbiota [125,126], whereas cervical and urethral environments present distinct physicochemical and immunological profiles. These differences directly influence retention, diffusion, and stability of phage-based therapeutics and should be explicitly considered in delivery system design [111,112,131].

In addition, physiological and exposure-related factors including menstrual cycle-associated hormonal fluctuations, semen exposure, and inflammation-induced changes in mucus composition may further influence local pH, barrier properties, and the retention and activity of phage-based therapeutics. These variables introduce temporal and host-specific heterogeneity that is rarely captured in preclinical models but is likely to be critical for clinical performance [111,112,125,126].

The mucosal environment imposes additional constraints on stability and activity. The vaginal niche is characterised by acidic pH and enzymatic activity, both of which can reduce the stability of phage particles and phage-derived proteins. Experimental evidence suggests that many bacteriophages exhibit optimal stability near neutral pH, with reduced viability under acidic conditions [87]. Mucus may also function either as a barrier or as a reservoir, depending on particle surface properties, further emphasising the importance of rational formulation design [131].

Host immune responses represent another important and incompletely understood constraint. Phage administration can induce both innate and adaptive immune responses, including the development of neutralising antibodies. These responses may persist following treatment, although their impact on therapeutic efficacy appears to vary depending on route of administration and duration of exposure [87,88,135].

Translational development of phage-based therapeutics for genital tract infections also presents important manufacturing, formulation and regulatory challenges. Reproducible large-scale production requires strict control of batch consistency, purity, and stability, particularly for biologically complex products such as live or engineered bacteriophages [12,81,136]. Endotoxin contamination derived from Gram-negative bacterial hosts remains a major concern and necessitates rigorous purification and quality-control procedures [81,90]. Regulatory classification may also differ substantially between therapeutic platforms, as live phages, genetically engineered phages, and purified phage-derived enzymes are likely to be evaluated under distinct regulatory frameworks with different safety and manufacturing requirements [90,136,137]. Furthermore, clinical evaluation in sexually transmitted infections presents additional complexities related to anatomical site heterogeneity, local tolerability, microbiome preservation, and the selection of clinically meaningful endpoints [111,112,131]. Together, these factors highlight that successful clinical translation will depend not only on antibacterial efficacy, but also on standardized manufacturing, formulation robustness, and carefully designed clinical development pathways [12,138].

Although clinically validated phage-based therapeutics for major STI-associated pathogens are still lacking, experience from other infectious disease settings provides useful clinical and developmental insights. Clinical and compassionate-use applications in non-STIs have demonstrated generally favourable safety profiles and in some cases, therapeutic benefit [12,139]. In addition, engineered phage therapy has shown clinical potential in difficult-to-treat multidrug-resistant infections [138]. At the same time, such findings cannot be directly extrapolated to STI-associated pathogens, where intracellular localisation, mucosal barriers, and structural constraints substantially alter accessibility.

The host microbiome represents an additional and often underappreciated factor influencing long-term therapeutic stability and ecological outcomes. Phage-derived therapeutics offer the potential for selective targeting, which may reduce disruption of commensal microbiota compared to broad-spectrum antibiotics [12]. However, evidence from broader microbiome studies suggests that phage–microbiome interactions can alter microbial community structure and ecological dynamics [140,141,142], indicating that selectivity should be evaluated within an ecological rather than purely pathogen-centred framework. In polymicrobial conditions such as bacterial vaginosis, pathogen eradication alone is unlikely to ensure durable clinical resolution. Recurrence is strongly associated with failure to restore a stable Lactobacillus-dominated microbial community [66]. This suggests that ecological imbalance, rather than the presence of a single pathogen, plays a central role in disease persistence.

Accordingly, phage-based strategies may need to be integrated into microbiome-aware therapeutic frameworks. Combination approaches such as co-administration with probiotics, ecological modulation, or sequential therapies targeting both biofilm disruption and microbiome restoration may be required to achieve sustained clinical benefit.

A major limitation remains the absence of clinically validated phage-based therapeutics for key bacterial STI pathogens, including *Chlamydia trachomatis*, *Neisseria gonorrhoeae*, *Mycoplasma genitalium*, and *Treponema pallidum*.

Among sexually associated conditions, bacterial vaginosis represents a partial exception. Phage-derived enzymes, including the engineered endolysin PM-477, have demonstrated activity in experimental and ex vivo biofilm models. However, even in this context, clinical validation remains limited, and recurrence remains a major challenge due to the polymicrobial and ecologically driven nature of the condition.

Stability and formulation challenges are particularly relevant for phage-derived enzymes such as endolysins and depolymerases. These biologics may lose activity due to proteolytic degradation, unfavourable physicochemical conditions, or insufficient residence time, and therefore often require stabilisation strategies such as encapsulation, protein engineering, or controlled-release systems [73,94,131,133,134]. These considerations reinforce the importance of integrating manufacturing, regulatory and formulation considerations into early-stage therapeutic development. The major pathogen-specific biological constraints, corresponding phage-related strategies, evidence maturity, and estimated translational readiness are summarised in Table 3.

Overall, progress in this field will depend on improved delivery systems, physiologically relevant models and microbiome-aware combination strategies. Extracellular and biofilm-associated conditions represent the most immediately tractable targets, whereas intracellular and structurally atypical pathogens remain substantially more challenging. Future work should prioritise physiologically relevant models, optimized mucosal retention strategies and microbiome-aware combination approaches. Without addressing these interconnected biological, technological, and regulatory constraints, the clinical potential of phage-based therapeutics is likely to remain limited despite significant advances in the field.

## 6. Conclusions

Bacterial sexually transmitted and sexually associated infections represent a biologically diverse group of conditions that differ in pathogen structure, infection niche, and accessibility to antimicrobial intervention. This diversity has direct implications for the development of phage-based therapeutics.

From this perspective, the limitations of phage-based approaches are inherently pathogen-specific. In *Chlamydia trachomatis*, intracellular localisation restricts access of extracellular agents. In *Mycoplasma genitalium*, the absence of peptidoglycan eliminates canonical targets for endolysin-based strategies. In *Neisseria gonorrhoeae*, outer membrane exclusion and antigenic variability complicate reliable targeting. In contrast, biofilm-associated bacterial vaginosis represents a setting in which the primary barrier is extracellular and therefore directly accessible to enzymatic disruption.

These differences highlight the need to move beyond generalised assumptions about phage therapy and toward pathogen-adapted strategies. Future progress is likely to depend less on the discovery of naturally occurring lytic phages and more on the development of engineered and functionally adapted platforms. Phage-derived enzymes, intracellular delivery systems, and receptor-targeted constructs may represent more realistic translational pathways than classical lytic phage therapy in several STI-associated pathogen contexts.

At the same time, several key challenges remain, including effective mucosal delivery, stability under physiological conditions, and the impact of host immune responses on therapeutic activity.

Future research should prioritise the development of site-specific delivery systems adapted to mucosal environments, validation of phage-based strategies in physiologically relevant in vitro and ex vivo models, and integration of phage-derived approaches with microbiome restoration strategies. In addition, the establishment of standardised translational and regulatory frameworks will be essential to enable clinical implementation.

Overall, phage-based intervention in bacterial sexually transmitted infections should be regarded as a biologically constrained but scientifically promising field. Accordingly, the future of phage-based therapeutics in this area will depend not on expanding the range of available agents, but on achieving effective integration of mechanism, delivery, and biological context.

## Figures and Tables

**Table 3:** Pathogen-specific translational barriers, phage-related therapeutic strategies, evidence maturity, and estimated translational readiness in STI-associated infections.

Pathogen	Key Translational Barrier	Most Plausible Phage-Related Strategy	Evidence Level	Translational Readiness
*Chlamydia trachomatis*	Intracellular localisation limits phage access	Nanoparticle-assisted intracellular delivery; engineered targeting systems	Conceptual	Low
*Neisseria gonorrhoeae*	Receptor variability and outer membrane accessibility	Engineered phages; phage-derived targeting platforms	Proof-of-concept	Low-Moderate
*Mycoplasma genitalium*	Absence of peptidoglycan cell wall	CRISPR-Cas-based targeting concepts; alternative intracellular strategies	Conceptual	Low
*Treponema pallidum*	Experimental inaccessibility and limited tractability	Future engineered or delivery-oriented phage-related approaches	Conceptual	Highly speculative
Bacterial vaginosis (*Gardnerella*-associated)	Extracellular polymicrobial biofilm environment	Endolysins; depolymerases; biofilm-disrupting enzymes	Experimental (preclinical)	Moderate (preclinical)

## Data Availability

No new data were created or analyzed in this study.

## Abbreviations

The following abbreviations are used in this manuscript:

AMR	Antimicrobial resistance
STI	Sexually transmitted infection
STIs	Sexually transmitted infections
BV	Bacterial vaginosis
WHO	World Health Organization
CDC	Centers for Disease Control and Prevention
*C. trachomatis*	*Chlamydia trachomatis*
*N. gonorrhoeae*	*Neisseria gonorrhoeae*
*M. genitalium*	*Mycoplasma genitalium*
*T. pallidum*	*Treponema pallidum*
CRISPR	Clustered regularly interspaced short palindromic repeats
Cas	CRISPR-associated proteins
DNA	Deoxyribonucleic acid
RNA	Ribonucleic acid
PM-477	Engineered endolysin targeting *Gardnerella* spp.
